# Copper and light shape a coastal picophytoplankton community via their combined effects on growth limitation and toxicity

**DOI:** 10.1093/ismeco/ycag124

**Published:** 2026-06-22

**Authors:** Katherine R M Mackey, Seth G John, Joana Tavares, Shun-Chung Yang, Kyeong Pil Kong

**Affiliations:** Earth System Science, University of California Irvine, Irvine, CA 92697, United States; Ecology and Evolutionary Biology, University of California Irvine, Irvine, CA 92697, United States; Earth Sciences, University of Southern California, Los Angeles, CA 90089, United States; Earth System Science, University of California Irvine, Irvine, CA 92697, United States; Earth Sciences, University of Southern California, Los Angeles, CA 90089, United States; Earth Sciences, University of Southern California, Los Angeles, CA 90089, United States

**Keywords:** copper, light, *Prochlorococcus*, *Synechococcus*, picoeukaryote, picophytoplankton, toxicity, growth, coastal, limitation

## Abstract

Copper (Cu) and light are resources that limit phytoplankton growth at very low (deficiency) or very high (toxicity) levels. The interactive effects of Cu and light on a coastal California picophytoplankton community were assessed during four incubation experiments using a 7 × 7 matrix of overlapping Cu and light gradients. Consistent with prior knowledge, sensitivity to Cu was greatest in *Prochlorococcus*, followed by *Synechococcus*, and then picoeukaryotes. In September, *Prochlorococcus* abundance declined with Cu additions >6 nM, whereas *Synechococcus* showed a toxicity threshold at >10 nM added Cu. An unexpected environmental increase in ambient seawater Cu prior to the October experiments brought *Prochlorococcus* and *Synechococcus* close to their toxicity thresholds, potentiating their sensitivity to light during October experiments. Synergistic effects between Cu and light exacerbated toxicity in both taxa, suggesting saturation of shared physiological stress response pathways. Addition of 10 mM nitrate at the start of one experiment did not rescue populations from this synergistic toxicity, e.g. by allowing de novo stress response enzyme synthesis. Across all experiments, picoeukaryotes were more resilient to high light and Cu, allowing them to persist or increase under conditions that limited *Prochlorococcus* and *Synechococcus*. This robustness combined with relief from resource competition upon decline of the other two taxa, ultimately led picoeukaryotes to dominate the communities despite having very low baseline relative abundances. Selection for different *Synechococcus* clades and picoeukaryote species likely permitted each of these populations to thrive over a broader range of Cu and light combinations than would be possible for populations with lower diversity.

## Introduction

Competition within phytoplankton communities is strongly influenced by the availability of growth-limiting resources. Copper (Cu) and light are two well-characterized resources that can limit phytoplankton growth at very low (deficient) or very high (toxic) levels. Cu is a cofactor for critical metalloenzymes, including plastocyanin, cytochrome oxidase, antioxidant enzymes, and multicopper ferroxidase [1, 2]. Free Cu (Cu′) is taken up intracellularly via active transport in a concentration dependent manner [3], and cells modulate [Cu′] in the surrounding seawater by producing strong extracellular ligands to avoid toxicity [4, 5]. Cu toxicity arises from reactive oxygen species (ROS) production, competitive inhibition of metal binding sites in metalloproteins, disruption of photosynthetic electron flow [6–8], and in *Synechococcus*, osmoregulatory-like responses [9]. Light is another essential resource required by all photosynthetic organisms. Under low light, cells lack energy to grow at optimal rates, whereas at high light, photosynthetic excitation pressure leads to photoinhibition [10–12]. Numerous photoacclimation strategies have evolved to optimize light harvesting and prevent photodamage [13].

Phytoplankton growth rates are responsive to nutrient and light levels in a concentration dependent manner. Nutrient uptake, including trace metal micronutrients, follows Michaelis–Menten kinetics, increasing linearly with concentration until a saturating concentration is reached [14]. Mirroring this relationship is the Monod equation relating growth rate and nutrient concentration, which follows a similar hyperbolic curve [15]. Early photosynthesis-irradiance curves modeled the effects of light using the same mathematical principles [11, 12], but were later modified to include a term for photoinhibition: the reduction in photosynthetic rates under high irradiance resulting from photodamage and other factors [16]. Hence, growth limitation due to resource deficiency and excess have long been recognized as important factors influencing the health and survivability of phytoplankton.

The combined interactive effects of more than one resource or environmental factor on growth rate is more complex. Growth responses to “multi-stressors” can be additive, synergistic, (greater than additive), or antagonistic (less than additive). The importance of multi-stressor experiments in studying the impacts of climate change have been emphasized [17–19], with many new studies on ocean acidification, warming-induced stratification, and pollution taking the multi-stressor approach [20–26].

This study sought to examine the potential interactive effects of Cu and light availability on a coastal marine picophytoplankton community in which resource scarcity and excess could both negatively affect community members. We conducted the study in the Southern California Bight during late summer and fall, when *Synechococcus, Prochlorococcus*, and picoeukaryotes are abundant [27–30]. Our goal was to determine the extent to which the combined effects of Cu and light influence growth and acclimation among the three groups, and contribute toward shaping picophytoplankton communities.

## Materials and methods

### Experimental site and incubation setup

This study sought to examine the potential interactive effects of resource availability on a simple marine microbial community when resource scarcity and excess can both negatively affect the growth of community members. We conducted the study in the Southern California Bight during the end of summer and fall, where the phytoplankton population has the greatest relative abundance of picophytoplankton, including *Synechococcus, Prochlorococcus*, and picoeukaryotes [27–30]. The dominance of picoplankton at this site is linked to prolonged stratification and declining surface N availability throughout the summer, with a corresponding reduction in the abundance of larger-celled taxa during the summer and fall [28], and intrusion of oligotrophic waters and populations from offshore [31]. During the stratified period, light levels decline and N concentrations increase with depth.

A total of four incubation experiments were conducted in the Southern California Bight during the late summer when surface waters are generally warmest and lowest in nitrogen (NO_3_^−^) [28]. Seawater for experiments A and B were collected on September 1, 2020 and seawater for experiments C and D were collected on October 22, 2020 ~ 5 km offshore of Newport Beach, CA (33.5649^o^N, −117.9567°E). Seawater was collected at midday using a sampling rosette, and was dispensed into acid cleaned, sample rinsed plastic carboys. Experiments A, C, and D used surface seawater, and experiment B used seawater collected from the rising limb of the deep chlorophyll maximum layer (DCM, ~20 m, ~4.3% PAR, Fig. S1) as determined from real time CTD fluorescence profiles. In September, experiment A tested the community acclimatized to higher light and lower NO_3_^−^ availability, whereas experiment B tested the community acclimatized to lower light and higher NO_3_^−^ availability at the same site and date. In October, surface water used in experiment D was identical to the surface seawater used in experiment C, except that the water was also amended with an additional 10 mM of chelexed NaNO_3_^−^ to test if relief of NO3- limitation affected sensitivity to Cu or light. (Depth profiles of temperature, salinity, chlorophyll fluorescence, and photosynthetically active radiation (PAR) for each date are included in the SI and Fig. S1).

Seawater was dispensed through a 20 mm mesh into 53 250 ml acid cleaned, sample rinsed polycarbonate bottles. Four were immediately processed to characterize baseline conditions. The remaining 49 bottles were separated into groups of seven, each receiving a spike of CuSO_4_ solution complexed with equimolar Na_2_EDTA to achieve different Cu′ concentrations of 0, 0.6, 1, 6, 10, and 60 nM Cu′ above background. The bottles were distributed into 7 submerged crates within an outdoor flowing seawater incubation tank, each with a different amount of screening resulting in 14, 16, 26, 37, 62, and 84% of ambient natural sunlight. A no-attenuation, 100% irradiance treatment was included. The setup resulted in 49 unique combinations of [Cu′] and light levels in a 7 × 7 treatment matrix. Bottles were incubated for 48 h. We note that nanoflagellates and small ciliate grazers would not be excluded by the 20 mm mesh prefilter and, if present, could have grazed preferentially on different picophytoplankton and affected their abundances in addition to the Cu and light treatments.

#### Nutrients

Concentrations of nitrate (NO_3_^−^), ammonium (NH_4_^+^), and phosphate (PO₄^3−^) were analyzed on a flow injection autoanalyzer (FIA, Lachat Instruments, Zellweger Analytics, Inc., QuikChem 8500 Series 2) following filtration (0.22 mm PES). Standards were prepared in Milli-Q water and blanks were prepared from aged, low nutrient seawater. The limit of quantitation (LOQ) was 0.2 μM (NO_3_^−^ and NH_4_^+^), and 0.1 μM PO₄^3−^.

#### Trace metals

Trace metal concentrations (Al, Cd, Co, Cu, Fe, Mn, Ni, Pb, V, and Zn) were determined on four baseline samples in each experiment. Using acid-cleaned plastic syringes and sterile 0.22 mm PES filter cartridges in a HEPA-filtered, plastic laminar flow hood, 30 ml of sample water was collected into acid-cleaned HDPE collection tubes.

Samples were acidified with concentrated, distilled, low trace metal hydrochloric acid at a sample-to-acid ratio of 1000:1 v/v. Acidified samples were stored in the dark for two weeks to recover labile particulate metals and dissolved metals adsorbed onto vial walls. Dissolvable trace metals were then measured in the PLASMA Facility at the University of Southern California. 15 ml sample was transferred to an acid-washed polypropylene Falcon tube (VWR; Catalog #89049–172), then 50 μl of an isotope spike (containing ^57^Fe, ^62^Ni, ^65^Cu, ^67^Zn, ^207^Pb, and ^110^Cd) was added. Samples incubated overnight before being preconcentrated by a SC-DX seaFAST system (Elemental Scientific). In the SeaFAST, ~10 ml of seawater was injected through the Nobias PA-1 column and 0.5 ml eluent (1 M HNO₃ containing 1 ppb In) was used to elute trace metals for concentration measurement.

Preconcentrated samples were analyzed using an Agilent 8900 Triple Quad ICP-MS tuned to achieve the highest sensitivities for ^57^Fe of 10 ppb Fe, ^115^In of 10 ppb In and ^208^Pb of 10 ppb Pb under MS/MS mode without using collision gas using the auto-tune function of the MassHunter Workstation. As the sensitivities were optimized, 2.0–2.5 ml min^−1^ of hydrogen (H_2_) gas was introduced into the collision cell to reduce the isobaric interferences on the elements of interest. The samples and a 10 ppb multi-element standard solution (Santa Clarita) were measured for their ^27^Al, ^110^Cd, ^114^Cd, ^59^Co, ^63^Cu, ^65^Cu, ^56^Fe, ^57^Fe, ^55^Mn, ^60^Ni, ^62^Ni, ^207^Pb, ^208^Pb, ^51^V, ^66^Zn, ^67^Zn, ^118^Sn, and ^115^In signals under the MS/MS H2 mode. The samples’ Cd, Cu, Fe, Ni, Pb, and Zn concentrations were obtained by isotope dilution method, and Al, Co, Mn, V concentrations were obtained by calibrating with In internally and with the 10 ppb multi-element standards externally.

The method’s LOQs were: dAl = 0.74 nM, dCd = 0.002 nM, dCo = 0.16 nM, dCu = 0.22 nM, dFe = 1,23 nM, dMn = 0.04 nM, dNi = 0.18 nM, dPb = 0.004 nM, dV = 0.10 nM, dZn = 0.16 nM. For use in calculating averages and statistical tests, measurements below LOQ were treated as LOQ divided by 2 for calculation purposes. Significance of differences between samples is reported after Bonferroni correction for a = 0.05 with two comparisons (i.e. experiment A × experiment B, and experiment A × experiments C, D), yielding an adjusted a-value for the comparisons of 0.025.

#### Flow cytometry

Flow cytometry samples were preserved with 4% formalin and analyzed at the Center for Aquatic Cytometry, Bigelow Laboratory for Ocean Sciences on a Bio-Rad ZE5 with 405 nm, 488 nm, and 640 nm lasers activated. Samples were thawed to 4°C and processed in fluorescence trigger mode using chlorophyll fluorescence (692/80 nm filter). In certain cases, *Prochlorococcus* gating had higher uncertainty due to dimness or low abundances. These data were retained for analysis because low abundance and dimness are expected potential outcomes of high light and high Cu exposure for *Prochlorococcus.* A single outlier (experiment C, *Prochlorococcus* at 100% light and 100 nM Cu) was excluded from analysis on the basis that it was an outlier that was >2 × SD greater than the mean.

#### Seawater back trajectory analysis

Back trajectory simulations were generated using the Lagrangian modeling framework OceanParcels v3.0.4^32^ for 60 days preceding sampling, with 25 particles spread 0.04 degrees apart near the sampling location advected in reverse time mode with a time step of 1 day using the Runge–Kutta fourth-order integration scheme. Surface geostrophic eastward and northward seawater velocities were obtained from the Global Ocean Gridded L4 Sea Surface Heights and Derived Variables Reprocessed 1993 Ongoing product (see “Data availability” section).

#### Multiple linear regression

Multiple linear regression (MLR) was used to characterize the independent and interactive effects of Cu and light on the abundances of *Synechococcus, Prochlorococcus*, and picoeukaryotes (see Supplemental Methods section for details in MLR terms and analysis). “Main effects” of Cu were determined by averaging the measured cell concentrations across all levels of light (and vice versa for the main effects of light). Main effects were identified as either linear, logarithmic, or quadratic by determining which relationship had the best fit to the data (highest R [2], Table S1). The independent variables were transformed accordingly based on those fits for the MLR. The interaction term was calculated as the product of the two transformed independent variables. Dependent variables (i.e. cell concentrations) were not transformed. Data were centered on their population means to reduce effects of collinearity, and scaled to their population standard deviations by calculating z-scores. The z-scores for each independent variable, the dependent variable, and the interaction term were used in the MLR analysis. To determine which, if any, of the individual or interactive effects was significant, *P*-values of the regression coefficients were used (a = 0.05) for regressions in which the significance *F* was <0.05.

## Results and discussion

Analysis of seawater chemical characteristics in the September and October experiments showed that the water column remained stratified during the two sampling periods, with a surface mixed layer depth of ~10 m, low NO3- concentrations, and similar chlorophyll *a* fluorescence levels (Fig. S1, Table 1) all consistent with typical seasonal patterns in the region [28]. Dissolved copper concentrations were 2.81 ± 0.08 nM in September experiment A surface water, 2.07 ± 0.16 nM in September experiment B DCM water, and 46.37 ± 0.2 nM in October experiments C and D surface water. Concentrations of other dissolved trace mentals are reported in Table 1.

**Table 1:** Characterization of baseline experimental seawater before treatments were made, showing nutrient concentrations (mM), dissolved trace metal concentrations (nM), and composition of initial phytoplankton cell concentrations (1000 × cells/ml). Data are reported showing mean ± SE. For use in calculating averages, individual measurements below LOQ were treated as LOQ divided by 2. In cases where the average of all measurements was below the LOQ, the LOQ is shown. The LOQ for each metal is given below its header in the table. The p-values for *t-*tests comparing background seawater metal concentrations between experiment are given in the final two rows, with a Bonferroni-corrected a value of <0.025 considered significant (bolded values). Percent of total picophytoplankton community is shown in parentheses for phytoplankton taxa.

	NO_3_ ^−^ (mM)	PO_4_ ^3−^ (mM)	NH_4_ ^+^ (mM)	NO_3_ ^−^:PO_4_ ^3−^ (−)
Experiment A	<0.20	0.13 ±0.01	0.33 ± 0.04	0.75	
Experiment B	1.19 ± 0.06	0.59 ± 0.01	0.28 ± 0.13	2.04	
Experiment C, D	<0.20	0.15 ±0.06	0.38 ± 0.48	0.67	
	Picoeukaryotes (x10^3^ cells/mL)	*Synechococcus* (x10^3^ cells/mL)	*Prochlorococcus* (x10^3^ cells/mL)		
Experiment A	12.4 ± 0.2 (9%)	55.2 ± 0.3 (41%)	68 ± 0.8 (50%)		
Experiment B	9.7 ± 0.1 (15%)	42.6 ± 0.3 (65%)	13.1 ± 1.5 (20%)		
Experiment C	6.1 ± 0.1 (6%)	61.5 ± 0.4 (62%)	31.8 ± 0.4 (32%)		
Experiment D	4 ± 0.8 (5%)	58.3 ± 1.1 (68%)	23.1 ± 1.4 (27%)		
	dAl	dCd	dCo	dCu	dFe
	(nM)	(nM)	(nM)	(nM)	(nM)
LoQ	0.30	0.03	0.12	0.82	3.35
Experiment A	4.97 ± 0.57	0.07 ± 000	<0.12	2.81 ± 0.08	4.69 ± 0.65
Experiment B	6.32 ± 0.46	0.11 ± 0.01	<0.12	2.07 ± 0.16	3.41 ± 0.73
Experiment C, D	4.64 ± 0.67	0.04 ± 0.01	<0.12	46.37 ± 0.2	42.84 ± 0.87
t-test (A x B)	0.126	**0.002**	NA	**>0.001**	0.149
t-test (A x C, D)	0.685	**>0.001**	NA	**>0.001**	**>0.001**
	dMn	dNi	dPb	dV	dZn
	(nM)	(nM)	(nM)	(nM)	(nM)
LoQ	0.08	0.23	0.03	0.34	1.39
Experiment A	0.18 ± 0.07	3.36 ± 0.07	0.11 ± 0.00	26.22 ± 0.31	92.7 ± 6.92
Experiment B	0.31 ± 0.03	3.45 ± 0.02	0.14 ± 0.01	25.96 ± 0.39	65.31 ± 6.23
Experiment C, D	1.65 ± 0.08	4.36 ± 0.05	0.99 ± 0.01	26.2 ± 0.35	121.23 ± 11.83
t-test (A x B)	0.249	0.347	**0.016**	0.652	0.061
t-test (A x C, D)	**>0.001**	**>0.001**	**>0.001**	0.969	**0.009**

**Figure 1:**
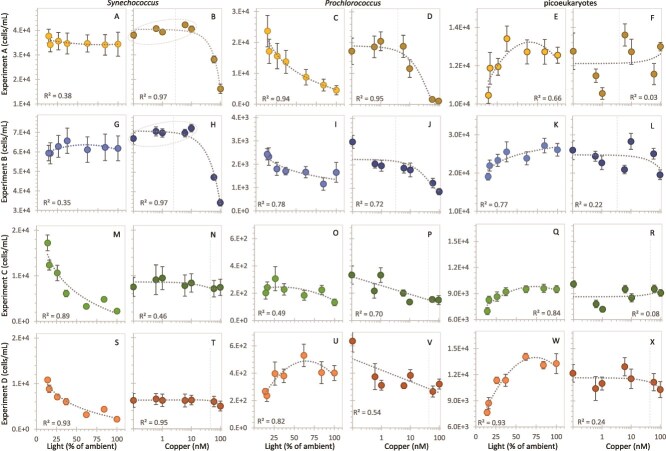
Main effect relationships for Cu′ and light for *Synechococcus* (left columns), *Prochlorococcus* (middle columns), and picoeukaryotes (right columns) in experiments A, B, C, and D (panels in rows 1–4, respectively). For each panel, data points show the average and standard deviation of all seven cell concentration measurements at each indicated Cu′ or light level. For example, in plots showing Cu′ concentration responses, the data point at a given Cu′ concentration is the average of all seven light level samples that received that Cu′ concentration. The best fit curve types used in each panel (dotted lines) are either linear, quadratic, or logarithmic (see Table S1), and were selected based on which curve type yielded the highest R^2^ value (lower left of each panel). Dashed grey vertical lines indicate the concentration of Cu′ in the initial baseline seawater before any Cu′ treatment additions were made. Grey circled regions in (b, h) indicate concentrations of potential relief from Cu′ limitation.

**Figure 2:**
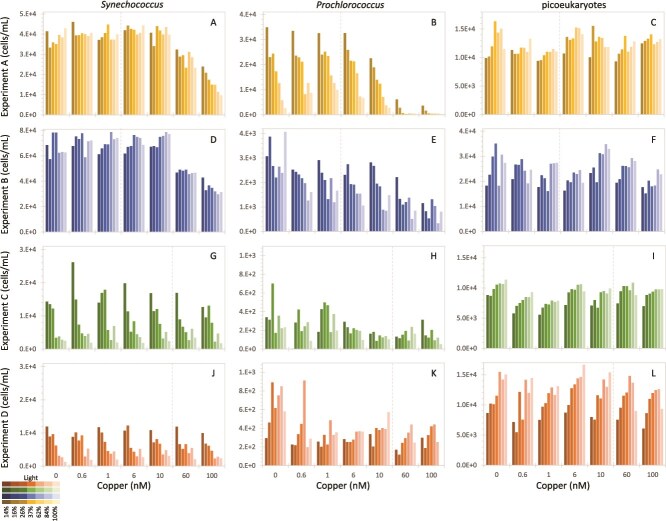
The simple effects of Cu and light on *Synechococcus* (left column), *Prochlorococcus* (middle column), and picoeukaryotes (right column) in experiments A (row 1), B (row 2), C (row 3), and D (row 4). Data collected at the seven light levels are plotted for each concentration of Cu, with the lowest light treatment shown in the darkest shade, and the highest light treatment shown in the lightest shade (color scale at lower left). Values to the right of the dashed grey vertical lines indicate additions that were higher than the background seawater Cu concentration to which cells were acclimatized at the time of collection (before Cu addition treatments were made), while those to the left were lower.

Trace metal and back trajectory analyses and indicate that surface seawater concentrations of Cu, Fe, Mn, Ni, Pb, and Zn were all significantly elevated in October compared to September, likely resulting from accumulation of terrestrial and sedimentary material that accumulated in the water following rain events and coastal mixing as surface currents hugged the coastline preceding October sampling (see supplemental information for expanded discussion). Consistent with these higher background metal concentrations, lower Cu treatment additions were needed to induce toxicity responses in *Prochlorococcus* and *Synechococcus* in October compared to September (Figs 1 and 2).

### Effect of copper on community composition

*Synechococcus, Prochlorococcus*, and picoeukaryotes are known to have a range of different requirements and tolerances for Cu, which drives their abundances in marine environments [9, 32]. While nutritive requirements for Cu among these taxa are varied due in part to the considerable microdiversity among ecotypes and strains of the same species, toxicity thresholds appear to follow a fairly consistent taxonomic trend of *Prochlorococcus* having the lowest Cu toxicity tolerance, followed by *Synechococcus*, and then picoeukaryotes [32–36].

**Figure 3:**
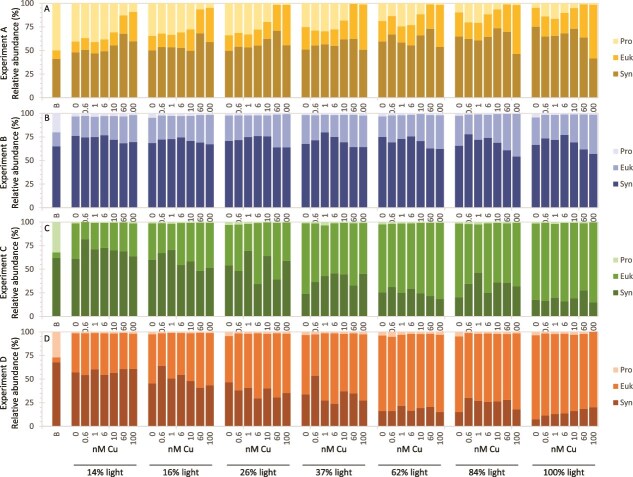
Relative abundances of *Synechococcus* (darkest shade), picoeukaryotes (middle shade), and *Prochlorococcus* (lightest shade) in (a) experiment A, (b) experiment B, (c) experiment C, and (d) experiment D. For each experiment, data are grouped first by light level, and then by order of increasing Cu concentration. Baseline abundances are marked “B” and are shown in the leftmost stacked bars.

Across the four experiments conducted in this study, *Prochlorococcus* consistently showed the greatest Cu toxicity sensitivity of the three taxa. In the September experiments A and B, *Prochlorococcus* abundance was suppressed at lower Cu levels (>6 nM) than for *Synechococcus* (>10 nM) or picoeukaryotes, where clear toxicity was not observed (Figs 1 and 2). Additionally, *Prochlorococcus* was nearly eliminated from the community at the two highest Cu additions (Fig. 3a).

**Figure 4:**
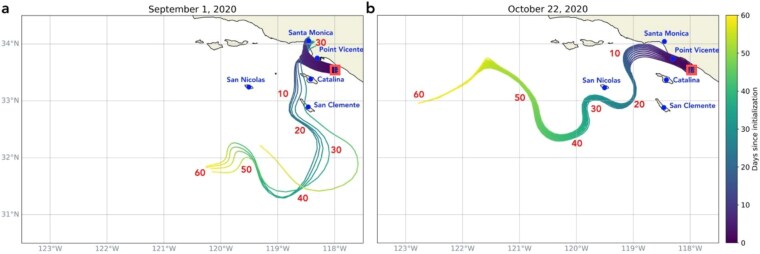
Surface water back trajectory simulations showing 60-day back trajectories of 25 particles released on (a) September 1, 2020 and (b) October 22, 2020 from the sampling site offshore of Newport Beach, CA. Days since the initiation of the simulation (red text) and geographical landmarks of note (blue text) are labeled. The red box shows the location of sample water collection for the experiments and indicates the starting grid box of the back trajectory.

Their higher sensitivity to Cu^33^ may be one reason that *Prochlorococcus* was much less abundant in the initial phytoplankton community in October experiments C and D (~30% of cells) compared to September experiment A (50%) (Table 1). Surface seawater collected in October for experiments C and D had concentrations of Cu, Fe, Mn, and Pb that were an order of magnitude higher than September in experiments A and B, as well as moderately elevated Ni and Zn (Table 1). The trace metal enrichment in October was likely due to urban runoff from two rain storms in the weeks preceding sampling and because the water parcel had travelled along the coastal margin for twice the distance in the same amount of time compared to September (Fig. 4), allowing the higher velocity water to entrain sediment via turbulence. The lower initial and final *Prochlorococcus* concentrations in experiments C and D therefore likely reflect a carryover toxicity effect from the higher background concentration of Cu in the October water (46 nM), which was already above the toxicity threshold observed in experiment A for *Prochlorococcus* (10 nM). The Cu additions made during experiments C and D would have further exacerbated the toxicity response, ultimately decreasing *Prochlorococcus* relative abundance to only a few percent by the end of the experiments (Fig. 3c, d).

*Synechococcus* abundance was only significantly affected by Cu additions in the September experiments. In both September experiments A and B, a sharp toxicity threshold was observed in which additions ≥60 nM led to toxicity, but additions ≤10 nM did not (Figs 1 and 2). This toxicity threshold was over an order of magnitude higher than the background concentration of Cu in the experiment water (2.8 nM, Table 1), suggesting that the *Synechococcus* population was not experiencing Cu toxicity stress at the time they were collected in September. In fact, a subtle enrichment effect for *Synechococcus* may be apparent following small Cu additions ≤10 nM in experiments A and B (Fig. 1b, h circled regions), and could suggest possible relief Cu limitation in this population. However, biological replication at each of the [Cu′] would be needed to determine if this effect is truly statistically significant.

*Synechococcus* abundance was not significantly affected by Cu additions in the October experiments C and D, likely because they were already acclimatized to elevated Cu when collected. The background concentration of Cu in the October seawater was elevated (46 nM, Table 1), falling between the concentrations that caused partial toxicity (60 nM) and subtle enrichment (10 nM) for *Synechococcus* in September experiment A. It is unlikely that this *Synechococcus* population experienced full toxicity before the experiments because the overall concentration of *Synechococcus* actually increased slightly from September to October, and came to represent 62–68% of the phytoplankton community (Table 1). Acclimatization of *Synechococcus* to higher Cu before experiments C and D would have provided sufficient time for cells to express their suite of Cu response genes in order to take advantage of Cu availability for metalloprotein synthesis, while also preventing damaging effects by synthesizing oxidative stress-related enzymes [4, 9, 37] and extracellular ligands to modulate the amount of free Cu′ in the surrounding seawater [4].

Another possibility is that different *Synechococcus* sub-populations, each with their own Cu requirements and tolerances, could have shifted in dominance between September and October 2020. An anomalous, late season *Synechococcus* bloom occurred in August 2016 in Southern California that reached atypically high abundances and was dominated by Clade II *Synechococcus* [30, 38], an open ocean group adapted to warm, oligotrophic conditions. *Synechococcus* in coastal California are typically dominated by Clades I and IV, which alternate in dominance throughout the year [29], and have specialized genomic islands that impart greater resistance to Cu stress [9, 39].

The seawater back trajectory analysis (Fig. 4) leaves open the possibility that the *Synechococcus* populations in our experiments comprised a mix of coastal and oceanic clades, with offshore Clade II cells entering coastal currents before mixing with the native community. In September, ~20% of the surface water originated offshore, and would have introduced a population of oceanic Clade II *Synechococcus* cells when it mixed with the coastal water parcel ~5 days before being collected for our experiments. The Cu sensitivity observed in September (Fig. 1b, h) is consistent with the population containing a higher proportion of less Cu-tolerant Clade II cells.

In October, intrusion of offshore water to the coast occurred much further upshore, north of Santa Monica Bay. This water mass hugged the coastline for 10 days before arriving at our sampling site, all the while accumulating metals from sediment and rainfall, which may have killed or weakened any Clade II *Synechococcus* that had mixed into the coastal population. Therefore, it is likely that a higher proportion of Cu-tolerant Clade IV cells would have survived the elevated Cu concentrations in seawater that emerged in October prior to experiments C and D, such that Cu sensitivity did not significantly affect their survival during our experiments (i.e. because they were already better adapted and acclimatized to higher Cu conditions) (Fig. 1n, t, Table 1). By whichever mechanism (acclimatization of the population via gene expression or a shift in the ratios of different clades within the population), the fact that toxicity was observed following 60 and 100 nM Cu additions in experiment A, but not in experiment C or D, is an *in-situ* example of Cu response adaptations directly influencing the survival of a natural *Synechococcus* population in the wild.

Consistent with prior observations from field and culture studies, picoeukaryotes were the most resilient of the three taxa to added Cu during our experiments [36, 40–42]. Cu additions independently did not have a significant effect on picoeukaryote abundance in any of the experiments (Table 2, Fig. 1) (although the interactive effect of Cu and light was significant in experiment D, discussed below.) Notably, the lack of significance for Cu stemmed from the considerable variability in picoeukaryote responses to Cu additions in all four experiments, which all showed very low R^2^ values (Fig. 1, Table S1). This variability could be due to the fact that picoeukaryote populations are functionally identified in flow cytometry based on their cellular fluorescence and size properties, but in reality comprise diverse assemblages of different species [43, 44], each with their own unique Cu requirements and toxicity thresholds [33, 43, 45, 46]. It is possible that the variability and lack of significance in picoeukaryote response to Cu that we observed reflects the net effect of combined positive and negative sub-population responses to added Cu.

**Table 2:** Multiple linear regression statistics, significance at *P* < 0.05 in bold.

Experiment	Statistic	Picoeukaryotes	*Synechococcus*	*Prochlorococcus*
Experiment A	Adjusted R^2^	0.0031	0.88	0.76
	Significance *F*	0.38	**<0.001**	**<0.001**
	*p*, Cu	0.37	**<0.001**	**<0.001**
	*p*, light	0.13	0.58	**<0.001**
	*p*, Cu x light	0.60	**0.0015**	**<0.001**
Experiment B	Adjusted R^2^	0.23	0.81	0.50
	Significance *F*	**0.0022**	**<0.001**	**<0.001**
	*p*, Cu	0.39	**<0.001**	**0.029**
	*p*, light	**0.012**	0.35	**<0.001**
	*p*, Cu x light	0.64	0.16	0.25
Experiment C	Adjusted R^2^	0.18	0.70	0.094
	Significance *F*	**0.0082**	**<0.001**	0.059
	*p*, Cu	0.15	0.15	0.029
	*p*, light	**0.0041**	**<0.001**	0.064
	*p*, Cu x light	0.56	0.20	0.30
Experiment D	Adjusted R^2^	0.40	0.78	0.22
	Significance *F*	**<0.001**	**<0.001**	**0.003**
	*p*, Cu	0.71	0.18	**0.014**
	*p*, light	**<0.001**	**<0.001**	0.13
	*p*, Cu x light	**0.046**	0.34	0.93

#### Effects of light intensity on community composition

The light intensity preferences of *Prochlorococcus, Synechococcus*, and picoeukaryotes share considerable overlap based on a range of factors from genetic adaptations (pigment type, antenna size, photoprotective mechanisms) to acclimatization status, which can modulate the aforementioned factors. In stratified water columns, surface phytoplankton populations can generally tolerate very high light along with periods of lower light that can occur due to episodic wind-driven mixing and cloud cover. Populations from deeper in the water column are acclimatized to lower light intensity and a more limited spectrum. Many sub-surface phytoplankton have the ability to tolerate exposure to high light for short time periods, but must possess the ability to acclimatize to high light if they are to survive under persistent high light surface conditions.

*Prochlorococcus* phylogeny is strongly influenced by light intensity, with high light (HL) and low light ecotypes each having distinct genetic fingerprints and photophysiological traits [47, 48]. These differences are conserved across different ocean basins and are a major niche-defining characteristic within the genus. While generally considered an open ocean organism, *Prochlorococcus* is a consistent member of the phytoplankton community in the Southern California Bight in the late summer through early fall [27, 28], with some cells persisting temporarily after surface current entrainment from the North Pacific Subtropical Gyre (NPSG) before winter cooling and mixing begins (Fig. 4).

In experiments A and B, high light intensity had a significant negative effect on *Prochlorococcus* abundance (Fig. 1c, i, Table S1). Compared to baseline populations, *Prochlorococcus* decreased 2- to 4-fold across treatments, with the largest declines occurring under high light (Fig. 2). This strong sensitivity to light is surprising, given that surface HL *Prochlorococcus* are well adapted to growth at surface irradiances. However, because this population is likely at least partially allochthonous [27, 49, 50], having likely originated in the NPSG (Fig. 4), it is possible that other aspects of the coastal environment unrelated to light are not optimal to support its growth in these waters. For example, exposure to a different compliment of trace metals could lower growth rates and increase susceptibility to photodamage in higher light, as could seasonal cooling within the Bight. *Prochlorococcus* in experiments C and D also appeared to follow this light sensitivity trend (Fig. 1o, u), although the effect was not significant due to noisiness introduced from the low fluorescence and abundances of *Prochlorococcus* in the initial seawater as discussed above.

The response of *Synechococcus* to light in these experiments was complex and changed over time. In September experiments A and B, initial surface and DCM populations had similar abundances (Table 1), and light treatment had no independent significant effect on growth (Table S1, Fig. 1a, g), even though cells in experiment B were acclimatized to light levels less than 10% of those in experiment A (Fig. S1). The flexibility of *Synechococcus* to grow over a wide range of irradiances is expected, given that strains of *Synechococcus* typically found in the Southern California Bight are known to possess genes for photoacclimation and photoprotection [51, 52]. However, sensitivity to high light was observed as an interactive effect with Cu in experiment A (Table 2), and had a significant independent negative effect on *Synechococcus* abundance in October experiments C and D, when background Cu concentrations in the seawater were already high (Fig. 1m, s). We discuss the implications of these interactive effects in the next section, below.

Light intensity was the experimental factor with the strongest influence on picoeukaryote abundance (Table 2). Across all experiments, the relationships between light intensity and picoeukaryote abundance took the shape of canonical photosynthesis-irradiance curves, with light limited growth apparent at the lowest irradiances, and reaching saturation at higher irradiances (Fig. 1e, k, q, w). Consistent with this observation, picoeukaryotes were more abundant in experiment A surface water than in experiment B at the DCM (Table 1).

### Interactive effects of Cu and light

One of the benefits of fully factorial experiments is that they permit the interactive effects between independent variables to be investigated. Quantifying interactive effects can help determine whether combinations of stressors cause greater than additive impacts on organisms and can help identify shared physiological response mechanisms. If two independent variables in a regression are both significant but their interactive effect is not, it indicates that they do not potentiate (nor mitigate) each other’s effects, and the combined stress effect is simply additive. Physiologically, it can signal that each variable affects the organism via a different physiological pathway or mechanism. Conversely, if an interactive effect is significant, it means that the two independent variables are synergistic (or antagonistic), and the combined effect of the two variables is greater (or less than) additive. Significant interactive effects can imply that shared physiological pathways are activated by each variable. If the combined variables saturate a shared stress response pathway, it can become overwhelmed and reduce the organism’s tolerance for each variable. Toxicity thresholds may be lowered under combined stress, making the organism more susceptible to harm at lower levels of toxin exposure.

In this study, the interactive effects of Cu and light were observed in three cases: *Prochlorococcus* in experiment A, *Synechococcus* in experiment A, and picoeukaryotes in experiment D (Table 2). Additionally, while not explicitly tested, an interactive effect was likely observed for *Synechococcus* in experiments C and D due to the (unanticipated) high background Cu concentration in the seawater prior to the experiments. Each of these scenarios is discussed below.

In experiment A, *Prochlorococcus* abundance was clearly influenced by light intensity and Cu concentration. The interactive effect of high Cu and high light is apparent where *Prochlorococcus* population declined to negligible levels lower than would be predicted for either variable independently (Fig. 2, in samples receiving Cu ≥60 nM and light ≥37%). The effect on overall community composition is striking in Fig. 3a, where the two highest Cu treatments have eliminated *Prochlorococcus* from the community. For *Synechococcus*, the interactive effect of Cu and light was significant and the individual effect of Cu alone was significant, but the individual effect of light was not (Table 2). *Synechococcus* concentrations only showed clear light inhibition in the highest Cu treatments (Fig. 2a). The low relative abundances of *Prochlorococcus* (2%) and *Synechococcus* (41%) resulted in picoeukaryotes coming to dominate the community in high Cu, high light treatments (57%, a substantial increase over their initial relative abundance of 9%), despite their relatively modest increase in absolute cell concentration (Fig. 2c).

The type of interactive effect observed for *Prochlorococcus* is an example of two stressors causing a synergistic effect that is greater than the sum of the two independent effects. This type of response could be due to a combination of unique and/or shared pathways being used to cope with Cu and light stress. However, the *Synechococcus* results imply that in order for light to affect *Synechococcus* abundance, the cells must already be stressed by Cu. This situation could arise if cells rely on the same stress response pathway to cope with both stressors.

Numerous stress response pathways have evolved for Cu and light, some of which are shared between the two stressors. For example, shared oxidative stress response mechanisms, like the production of superoxide dismutase (SOD) and other antioxidant enzymes, are important in protecting the cell from reactive (radical) oxygen species (ROS) that can be generated by both high light and Cu^2^. ROS production is an inevitable consequence of photosynthesis that results from elevated photosynthetic excitation pressure under high light, whereas Cu generates ROS directly through its own redox chemistry via the Haber-Weiss cycle [53], and can lead to photosynthetically-generated ROS by interfering with plastoquinone electron transport at the level of photosystem II [7, 8] in a manner that is potentiated by light [54]. Hence, cells may use SOD to cope with radicals generated from both light and Cu stress.

The observation that light alone was not a significant determinant of *Synechococcus* abundance in experiment A suggests that, ordinarily, these cells could cope with any ROS that were produced during photosynthesis if and when other photoprotective mechanisms fell short, possibly by relying on antioxidant pathways like the SOD pathway. However, in the presence of high Cu ROS, the dismutation capacity of cellular SOD could become saturated, thereby limiting the pathway’s ability to protect against additional, photosynthetically-derived ROS. Additionally, independent of the SOD pathway, Cu inhibition of photosynthetic electron transport can increase the amount of ROS production and physiological stress due to light, meaning that light exposure causes more ROS accumulation in the presence of inhibitory Cu than it does otherwise [54]. By either mechanism (direct or indirect effect of Cu on ROS production), the effect of phototoxicity in *Synechococcus* would only be revealed in the presence of high Cu; hence, the interactive effects of Cu and light become significant, even when light alone is not.

On the other hand, Cu and light stress responses can also manifest via independent mechanisms in the cell. For example, high light can activate photoprotective mechanisms that prevent photodamage from oxidative stress by down regulating photosystem II and light harvesting [55–57], and Cu does not directly induce to these mechanisms. However, Cu toxicity from competitive inhibition of metalloproteins, such as the substitution of Cu^2+^ for Mg^2+^ in chlorophyll reaction centers [58], is a process that can limit growth independent of light intensity and requires different internal detoxification strategies, like sequestration and precipitation [59]. The observation of Cu and light having significant interactive *and* independent effects, as observed for *Prochlorococcus*, could arise in organisms where Cu and light limit growth via multiple mechanisms; e.g. if light induces down regulation of light harvesting, Cu induces competitive inhibition, and both induce ROS formation.

While identifying the specific pathways that gave rise to the results observed here is outside the scope of the current study, these examples serve to illustrate how co-occurring environmental stressors with overlapping physiological responses could lead to synergistic effects greater than would be predicted based on single stressors alone. Interactive effects make toxicity thresholds appear lower than they are in isolation, so failing to account for their effects in wild populations could lead populations to appear more vulnerable than they might otherwise be under single stressor conditions. A good example of this was *Synechococcus* in experiments C and D, for which only light was identified as a significant variable influencing growth (Table 2), whereas Cu and interactive light and Cu (but not light alone) were significant in experiment A. In reality, the strong sensitivity of this *Synechococcus* population to light was likely due to the pre-existing high background Cu concentrations in the experiments C and D seawater, making it a *de facto* interactive effect more similar to the responses observed in experiment A for the high Cu addition treatments. Interpretation of how toxicity thresholds influence community competition in the wild should take particular care to consider all possible factors that affect cell growth and survival and that could confound observations in single stressor experiments.

The final case of a significant interactive effect for Cu and light occurred for picoeukaryotes in experiment D, for which the individual effect of light, but not Cu, was also significant. The picoeukaryote populations in experiments C and D increased 2- and 3-fold relative to baseline levels, but only the effect in experiment D was significant, likely due to the added NO_3_^−^ in experiment D allowing more growth. This resulted in the picoeukaryote population dominating the community at relative abundances >80% in the highest light treatments, despite being only a small fraction (5%) of the overall community in the baseline seawater (Fig. 3d).

The cause of the interactive role of Cu for picoeukaryotes is not clear, given that the relationship between Cu and abundance was not significant independently and had a low R^2^ value of 0.24 (Fig. 1x). One possibility is that the high background Cu concentrations, together with Cu and light treatments in the experiment, led to such sharp declines in *Prochlorococcus* and *Synechococcus* that the picoeukaryotes were released from competition for other growth limiting nutrients. Nitrogen limitation of picoeukaryotes in experiment D would have already been alleviated through the experimental addition of NO_3_^−^, but relief from competition may have facilitated acquisition of PO_4_^3−^, Fe, or other micronutrients. This indirect growth effect, acting via changes in the abundances of the other two picophytoplankton populations, could explain why an interactive effect was observed for Cu and light in picoeukaryotes, in that it is essentially a proxy for increased availability of another limiting resource(s). In other words, high Cu and light may benefit picoeukaryotes more than would be expected based on their physiology alone, because those stressors kill their competitors and thereby eliminate other growth obstacles, like competition for different shared resources.

### Other considerations affecting interacting stressor outcomes

Growth limitation from toxicity depends strongly on compensatory mechanisms to mitigate the effects of potential toxicants. Because most of these require synthesis of specialized enzymes, the cell’s ability to acclimate depends on N availability for protein synthesis. To determine if N availability affected phytoplankton responses to Cu and light, experiment D included 10 mM NO_3_^−^ enrichment, but was otherwise identical to experiment C. The response curves for *Prochlorococcus* and *Synechococcus* were very similar between experiments C and D (Fig. 1m–p, s–v), which could indicate that NO_3_^−^ availability does not help those cells acclimatize to light and Cu stress in this system. Moreover, many surface *Prochlorococcus* ecotypes are unable to use NO_3_^−^ [60], and so may have been unable to benefit from NO_3_^−^ additions in this study. More likely is that poor cell health from the high background Cu concentrations masked any beneficial effects of N in *Prochlorococcus* and *Synechococcus*. The main outcome of the NO_3_^−^ addition was picoeukaryote fertilization, which contributed to their dominance in experiment D. The ability of N to modulate toxicity by enhancing an organism’s acclimatization capacity warrants more investigation.

The final type of interactive effect that could be observed in multi-resource experiments is when growth limitation from scarcity of one resource intersects with growth limitation from toxicity of the other. Limitation from Cu scarcity was only observed for *Synechococcus* in experiments A and B (Fig. 1b, h, circled regions). While the significant interactive effect was apparent under toxicity-inducing high Cu and light as discussed above, it does not extend to samples receiving low Cu (starvation) together with high light (photoinhibition). Were this to have occurred, we would expect high light, Cu-starved samples to have lower than additive abundances, but this was not observed. Rather, *Synechococcus* in those high light, Cu-starved samples was at least as abundant (if not more so) as in low light, Cu-starved samples (Fig. 2a, compare the lightest bar to the darker ones for samples receiving ≤10 nM Cu). Therefore, Cu scarcity did not potentiate high light stress or contribute to the significant interactive effect for *Synechococcus* in this experiment (Table 2).

The types of interactions considered in this study could become more common in the future with warming-induced expansion of stratified oligotrophic regions dominated by picophytoplankton communities [61, 62]. In these regions, phytoplankton trapped in surface layers would be exposed to longer periods of full sun and accumulation of atmospheric deposition, potentially causing higher concentrations of many different metals simultaneously. Full sunlight and higher metal concentrations could also accompany persistent, intensified nutrient limitation due to reduced mixing, leading to potential combinations of (trace and macro) nutrient starvation, metal toxicity, and photoinhibition. This study highlights the importance of considering the interactive effects of multiple stressors when designing experiments to understand the effects of global change on marine microbial communities, particularly in terms of understanding their effect on competition among picophytoplankton taxa.

## Supplementary Material

Copper_and_light_field_experiments_Supplemental_20260329_ycag124

## Data Availability

This study used CMEMS Level 4, 0.125 degree SLA and geostrophic velocity gridded global ocean dataset, version 008_047 (https://doi.org/10.48670/moi-00148). The OceanParcels v. 3.0.4 Python package was used to conduct Lagrangian particle simulations [63]. Raw datasets used in this study were deposited in Dryad Database (DOI: 10.5061/dryad.3r2280gv8).
