# Supplementary material for: Copper and light shape a coastal picophytoplankton community via their combined effects on growth limitation and toxicity
Source: ISME Commun. 2026 Jun 22;6(1):ycag124. doi: 10.1093/ismeco/ycag124 (PMC13374865; doi:10.1093/ismeco/ycag124)
Supplement: Copper_and_light_field_experiments_Supplemental_20260329_ycag124 [file copper_and_light_field_experiments_supplemental_20260329_ycag124.docx]

***Multiple linear regression (MLR) statistical analyses.***

Multiple linear regression was used to characterize the independent and interactive effects of Cu and light levels on the abundances of *Synechococcus*, *Prochlorococcus*, and picoeukaryotes in each of the four experiments. “Simple effects” were characterized by examining the relationship between one independent variable and the dependent variable at a specific level of the other independent variable. For example, the effect of light level was examined for each Cu concentration, and vice versa. This resulted in 7 simple effect relationships per independent variable, per experiment, per species (7 independent variable treatment levels x 2 independent variables x 4 experiments x 3 species = 168 unique relationships total).

For each experiment and species, the “main effect” of each independent variable (Cu and light) was determined by averaging the measured values of the dependent variable (*Prochlorococcus*, *Synechococcus*, and picoeukaryote concentrations) across all levels of the other independent variable. For example, the main effect of light in any given experiment was determined by averaging the cell concentrations at each light level across all levels of Cu (and vice versa). Average values were plotted against each of their independent variables, resulting in two main effect graphs (one for light and one for Cu) for each experiment and taxa (24 total). The main effects of Cu and light on cell concentration were identified as being either linear, logarithmic (ln), and second-order polynomial (quadratic) by fitting curves and determining which relationship had the best fit (highest R^2^) for each plot (Table S1).

Interactive effects occur when the effect of one independent variable on the dependent variable is influenced by the values of the other independent variable(s). To determine if interactive effects between Cu and light occurred in the experiments, MLR was performed in Microsoft Excel for each experiment (A, B, C, and D) and taxa (*Synechococcus*, *Prochlorococcus*, and picoeukaryotes) independently. The input variables included terms for light, Cu, and the interactive effect of light and Cu (light*Cu). The independent variables were transformed based on the type of relationship identified in the main effect analyses, and were either squared (for polynomial best fit), ln transformed (for logarithmic best fit), or kept unchanged (for linear best fit) (Table S1). The interaction term was calculated as the product of the two transformed independent variables. Dependent variables (i.e., cell concentrations) were not transformed for the analysis.

Following the transformation step, all data were centered on their respective population means to reduce effects of collinearity, and scaled to their respective population standard deviations by calculating z-scores (i.e., the number of standard deviations a given data point lies above or below the population mean). For each experiment and species, z-scores were computed as [(transformed data value - population mean of the transformed data set)/ standard deviation of the transformed data set]. The z-scores for each independent variable, the dependent variable, and the interaction term were used in the regression analysis. To determine which, if any, of the individual or interactive effects was significant, *p*-values of the regression coefficients were used for regressions in which the significance *F* was < 0.05 (Table 2). Significance was assigned for coefficient *p*-values < 0.05. Adjusted R^2^ values, which tell the goodness of fit for the regression while accounting for independent variable predictors that are not significant in the regression model, are also reported.

**Results**

***Water column chemistry (CTD).*** Water column hydrographic characteristics were similar between the September and October sampling dates (Supplemental Figure S1). In September, the surface water temperature was 21.0^o^C, and the salinity was 33.64 psu. The peak chlorophyll concentration reached 13.3 μg/L at 27m depth, where irradiance was ~0.7% of the “total” irradiance at the air/water interface (i.e., at 0 m). Surface water from the upper 1 m of the water column collected for experiment A was attenuated to 67% of total, whereas at the depth of the rising limb of the DCM (20 m, where the water for experiment B was collected) was 4.3% of total.

In October, surface temperature (21.7^o^C) and salinity (33.63psu) were similar to September values, but the chlorophyll maximum had shoaled to 19m, reaching 9.82 μg/L, where irradiance was ~1.8% of the total irradiance at 0 m (Supplemental Figure S1). Surface water from the upper 1 m of the water column collected for experiments C and D was attenuated to 58% of total irradiance at 0 m. The base of the euphotic zone, taken as the depth at which PAR is attenuated to 1% of surface levels, was 25m in September and 22m in October.

***Surface seawater back trajectories*.** To identify the source locations of the water collected on the two experiment dates, back trajectory analysis was performed (Figure 4). In both cases, offshore Pacific Ocean surface water was entrained within the bight, entering the coastal area west of Catalina and San Clemente Islands, and east of San Nicholas Island. For the September simulation, 20% (5 of the 25 advected particles) of the trajectories originated in the offshore Pacific, and the other 80% (20 of the 25 particles advected) originated from within the Bight near Santa Monica Bay. The offshore water entered the Bight just west of Catalina and San Clemente Islands. This parcel then merged with the parcel from Santa Monica near Point Vicente, and they flowed southeast along the coast toward Newport Beach together for ~5 days.

For the October simulation, all of the trajectories originated in the offshore Pacific and entered the Bight midway between Catalina and San Clemente Islands to the east, and San Nicolas Island to the west. The water turned to the northwest before a hairpin turn around Thousand Oaks sent it southeast, hugging the coastline as it flowed for the final ~10 days toward Newport Beach. Overall, the October parcel covered twice the distance (~120 km) along the coast preceding experiments C and D compared to the September parcel in experiments A and B (~55 km).

**Baseline seawater characteristics**

***Nutrient concentrations.*** Nutrients in the experimental seawater had low NO_3_^‑^:PO_4_^3-^ ratios that were well below the Redfield Ratio of 16:1 (Table 1). NO_3_^-^ concentrations were below the LOQ in experiment A, C, and D surface water, and 1.19 ± 0.06 μM for experiment B (DCM water). PO_4_^3-^ was above the LOQ in all experiments, with experiment B having the highest level (0.59 ± 0.01 μM), and surface water experiments A, C, and D having lower levels (~0.15 μM). The NH_4_^+^ levels were likewise above the LOQ, though with less variability among experiments (~0.3-0.4 μM) (Table 1).

***Dissolved trace metal concentrations.*** Trace metals were analyzed for all of the seawater batches used in the experiments before any treatments were made and are shown in (Table 1). In September, surface water (5 m, experiment A) was significantly higher in Cu (p<0.001) and significantly lower in Cd (p=0.0020) and Pb (p=0.016) than DCM water (20 m, experiment B); whereas, there was no significant difference between surface and DCM concentrations of Al, Co, Fe, Mn, Ni, V, or Zn. Surface water from October (experiments C and D) had significantly higher concentrations of Cu (p<0.001), Fe (p<0.001), Mn (p<0.001), Ni (p<0.001), Pb (p<0.001), and Zn (p=0.009), and lower concentrations of Cd (p<0.001) than surface water from September (experiment A). There was no difference between Al, Co, or V concentrations in September and October surface samples.

There is high certainty that the elevated metal concentrations in the October water were of terrestrial or sediment origin, rather than sample contamination, because the compliment of metals that were elevated in the water (Mn, Fe, and Ni, Table 1) all have strong terrestrial and sedimentary sources that can cause their concentrations to covary^35^. Moreover, the main source of Mn to the ocean is benthic release from continental shelf sediments via reduction of Mn oxides coupled to organic matter degradation and sulfide production^35^, and accordingly, Mn contamination during sample processing is rare. Finally, Zn concentrations are typically present in very high concentrations relative to other metals in samples contaminated through sample processing. Zn levels in the October water were not abnormally elevated relative to the shelf sediment-derived metals, further confirming that sedimentary release was a major source of the elevated metals in October samples.

***Phytoplankton community composition.*** Enumeration of picoeukaryote, *Synechococcus*, and *Prochlorococcus* cell concentrations indicated that all four experiments began with each taxa represented at different relative abundances. The initial compositions of the phytoplankton communities were measured before any manipulations were made to the seawater (Table 1). *Synechococcus* ranged from 41-68%, *Prochlorococcus* ranged from 20-50%, and picoeukaryotes ranged from 5-15% of the initial communities across experiments. *Synechococcus* was the most abundant taxon in experiments B, C, and D (representing 62-68% of cells counted), whereas *Prochlorococcus* was most abundant in experiment A (50% of cells counted). Picoeukaryotes comprised a smaller fraction of the initial populations in all four experiments (5-15% of cells counted).

**Effects of Cu and light on phytoplankton abundances**

The MLR analysis tested for significance of Cu, light, and combined (Cu*light) main effects on phytoplankton cell concentrations after transforming each dataset according to the best fit trends (Table S1, Figure 1).

**Experiment A**

In experiment A, a threshold response in *Synechococcus* abundance was observed above the 10 nM Cu addition, where treatments at or below 10 nM Cu had similar *Synechococcus* abundances, and treatments receiving 60 nM and 100 nM Cu showed lower *Synechococcus* abundances (Figure 1B). *Synechococcus* abundances were less sensitive to light intensity (Figure 1A). The main effect best fit trends for *Synechococcus* were both quadratic for Cu (R^2^=0.97) and light (R^2^ = 0.38) (Table S1). The MLR was significant (F_sig_ < 0.001), R^2^_adj_ = 0.88), and showed the main effect of Cu (*p* < 0.001) and the interactive effect of Cu*light (*p* = 0.0015) were both significant, while the effect of light alone was not (Table 2). Inspection of the simple effects plots for *Synechococcus* only begin to show a consistent trend for light emerge at the highest Cu addition treatment of 100 nM, where a linear relationship between abundance and light attenuation was apparent (Figure 2A), suggesting that the combined effect of Cu and light may be more apparent toward higher Cu concentrations in the environment. Though not statistically significant, *Synechococcus* appeared to show a slight fertilization effect from Cu at 1 nM compared to samples with no added Cu (Figure 1B, circled region).

The main effect best fit trends for *Prochlorococcus* in experiment A were quadratic for Cu (R^2^=0.95) and logarithmic for light (R^2^ = 0.94) (Figure 1C,D, Table S1). The MLR was significant (F_sig_ < 0.001, R^2^_adj_ = 0.76), and showed that *Prochlorococcus* abundance was significantly affected by Cu (*p* < 0.001), light (*p* < 0.001), and the interaction of Cu*light (*p* < 0.001) (Table 2). The simple effect plots for *Prochlorococcus* show a strong effect of light, with higher abundances observed at lower light levels (Figure 2B). The simple effect plot also shows growth inhibition at high Cu additions ≥10 nM that was apparent across all light levels. However, a fertilization effect from low-level Cu additions was also apparent (though not statistically significant, as with *Synechococcus*), where the greatest *Prochlorococcus* abundances were observed for the 1 nM Cu addition (Figure 1D). Growth limitation from Cu scarcity was observed at lower Cu additions, particularly under the highest light treatments (Figure 2B). The combination of growth limitation at both low and high Cu addition levels under high light gives the Cu-abundance main effect relationship a parabolic shape (Figure 1D).

Picoeukaryotes showed much weaker responses to Cu and light compared to *Synechococcus* and *Prochlorococcus* at the simple and main effect levels (Figure 2C, Figure 1E,F). The main effect best fit trend was linear for Cu (R^2^ = 0.033) and quadratic for light (R^2^ = 0.66) (Table S1), but the MLR for picoeukaryotes in experiment A was not significant (F_sig_ = 0.38, Table 2).

**Experiment B**

In experiment B water from the DCM, *Synechococcus* abundance followed quadratic main effect trends for Cu (R^2^ = 0.97) and light (R^2^ = 0.35) (Figure 1G,H, Table S1). The MLR was significant (F_sig_ < 0.001, R^2^_adj_ = 0.81), and showed that the effect of Cu on *Synechococcus* abundance was significant (*p* < 0.001, Table 2). The simple effect plot shows a similar but sharper threshold response to Cu as for *Synechococcus* in experiment A; as in the surface population, these DCM *Synechococcus* had lower abundances at Cu addition treatments above 10 nM regardless of light level (Figure 2D).

*Prochlorococcus* was less abundant in the DCM community compared to the surface community (Table 1). *Prochlorococcus* abundances decreased with both increasing Cu and light, where the main effect best fit trend was quadratic for Cu (R^2^ = 0.72) and logarithmic for light (R^2^ = 0.78) (Figure 1I,J, Table S1). The MLR was significant (F_sig_ < 0.001, R^2^_adj_ = 0.50, Table 2), showing that the individual effects of light (*p* < 0.001) and Cu (*p* = 0.029) significantly affected *Prochlorococcus*, but the interactive effect of Cu*light did not (_p_ = 0.25). This is in contrast to the surface *Prochlorococcus* population from experiment A, where the interactive effects of Cu*light did significantly affect *Prochlorococcus* abundance. The simple effect plots for experiment B support the MLR results by showing the decreasing abundance trends with increasing Cu and light individually (Figure 2E). However, there is more noise in this dataset because the strength of the *Prochlorococcus* fluorescence decreased closer to its detection limit in experiment B (likely due to the cells being acclimated to the lower light of the DCM and bleaching during experimental exposure to higher light), making it more difficult to differentiate *Prochlorococcus* cells from other small, non-fluorescent particles in the water. The noisiness of the data may have precluded detection of interactive effects between Cu and light at statistically significant levels, even though Cu and light both individually affected *Prochlorococcus* levels significantly (Table 2).

The picoeukaryote abundance main effect best fit trend was linear for Cu (R^2^ = 0.22) and logarithmic for light (R^2^ = 0.77) (Figure 1K,L, Table S1). The MLR was significant (F_sig_ = 0.0022), though the overall variance in picoeukaryote abundance that was explained by light and Cu was low (R^2^_adj_ = 0.23, Table 2). Light had a significant effect, with higher picoeukaryote abundances observed for higher light levels (*p* = 0.012). The simple effect plot shows this effect of irradiance (Figure 2F). The simple effect plot also suggests a bimodal response to Cu additions in which two distinct populations appear to be present, one at low Cu levels that declines with abundance up to 6 nM of added Cu, and the other more abundant at higher Cu additions ≥10 nM.

**Experiments C and D.**

Because the initial seawater was collected from the same time, depth (surface), and location for experiments C and D, the initial seawater conditions were similar (Table 1), so any differences between the results of the two experiments is attributable to the 10 μM NO_3_^-^ spike that was added to the experiment D seawater at the very start of the experiment.

The *Synechococcus* populations had main effect best fit trends that were quadratic for Cu in experiment C (R^2^ = 0.46) and experiment D (R^2^ = 0.95), and logarithmic for light in experiment C (R^2^ = 0.89) and experiment D (R^2^ = 0.93) (Figure 1M,N,S,T, Table S1). The MLRs for experiments C (F_sig_ < 0.001, R^2^_adj_ = 0.70) and D (F_sig_ <0.001, R^2^_adj_ = 0.78) were significant, and showed that the effect of light on *Synechococcus* abundance was significant in both experiments (experiment C, *p* < 0.001; experiment D, *p* <0.001; Table 2), where abundance and light intensity were inversely related. *Synechococcus* abundances were not significantly affected by Cu addition or the interactive effect of Cu*light. The simple effects plots for *Synechococcus* in experiments C and D show the clear inverse relationship between light and *Synechococcus* abundance (Figure 2G,J). *Synechococcus* abundances were similar for high light treatments across Cu concentrations in experiments C and D; however, they were approximately twice as high in experiment C compared to experiment D under low light treatment, despite experiment D receiving added NO_3_^-^ (Figure 2G,J).

*Prochlorococcus* populations in experiments C and D declined sharply over the course of the experiments relative to baseline water, with some samples having *Prochlorococcus* fluorescence signals and abundances close to detection and reporting limits (see methods section). We discuss the analysis of these results here with the understanding that these populations represent a very small fraction of the overall phytoplankton community in the final samples from experiments C and D, and possible reasons for this observation are provided in the discussion.

The *Prochlorococcus* populations had main effect best fit trends that were logarithmic for Cu in experiment C (R^2^ = 0.70) and experiment D (R^2^ = 0.54), and quadratic for light in experiment C (R^2^ = 0.49) and experiment D (R^2^ = 0.83) (Figure 1O,P,U,V, Table S1). The MLR for experiments C (F_sig_= 0.059, R^2^_adj_ = 0.09) did not meet the cut off for significance at α = 0.05; however, had the F_sig_ for experiment C been significant, Cu would have had a significant main effect (*p* = 0.029) and light would have been close to significance (*p* = 0.064, Table 2), reflecting the general observation that high Cu and light samples had lower *Prochlorococcus* abundances (Figure 1O,P, Figure 2H,K). In experiment D, the MLR was significant (F_sig_ = 0.0030), R^2^_adj_=0.22), and Cu had a significant effect on *Prochlorococcus* abundance (*p* = 0.014, Table 2). The significant inverse relationship between Cu and *Prochlorococcus* abundance in experiment D (but not in C) is driven mainly by the higher abundances in samples with no added Cu (Figure 2K, i.e., *Prochlorococcus* in experiment D samples with 0 nM added Cu were 2- to 3-fold more abundant than in samples receiving added Cu), which could be due to the addition of NO_3_^-^ allowing greater survival at lower Cu in experiment D. There was no significant interactive effect of Cu*light on *Prochlorococcus* that could be identified in either experiment C or D. We note that interpretation of the lack of significance for light and the combined effects of Cu*light for *Prochlorococcus* in experiments C and D must take into account the very small population sizes that were near the method’s LOQ, and the associated loss of statistical power that may have masked these effects.

The picoeukaryotes had main effect best fit trends that were linear for Cu in experiment C (R^2^ = 0.078) and experiment D (R^2^ = 0.24), and quadratic for light in experiment C (R^2^ = 0.84) and experiment D (R^2^ = 0.93) (Figure 1Q,R,W,X, Table S1). The MLRs for experiments C (F_sig_ = 0.0082, R^2^_adj_ = 0.18) and D (F_sig_ < 0.001, R^2^_adj_ = 0.40) were significant, and showed that the effect of light on picoeukaryote abundance was significant in both experiments (experiment C, *p* = 0.0041; experiment D, *p* < 0.001). Additionally, the interactive effect of Cu*light was significant in experiment D (*p* = 0.046), but not experiment C (Table 2). Both experiments C and D show parabolic trends for picoeukaryote abundance in response to light that is similar to a traditional photosynthesis-irradiance curve; abundances increase with increasing light until a maximal level is reached, and above which they plateau (Figure 1Q,W). The effect is more pronounced in experiment D compared to experiment C, possibly because the addition of NO_3_^-^ in experiment D was able to support additional growth under the high light conditions that was not possible in experiment C, where NO_3_^-^ was scarce.

As in experiment B, picoeukaryotes in experiments C and D showed a somewhat bimodal response to added Cu (Figure 2I,L) that could be due to the presence of two or more subpopulations (e.g., different species) with different toxicity thresholds

Supplemental Table S1: Main effect transformations and correlation coefficients.

| Independent variable | Dependent variable | Experiment | Best fit type | R^2^ |
| --- | --- | --- | --- | --- |
| Cu | *Synechococcus* | A | quadratic | 0.97 |
| Cu | *Synechococcus* | B | quadratic | 0.97 |
| Cu | *Synechococcus* | C | quadratic | 0.46 |
| Cu | *Synechococcus* | D | quadratic | 0.95 |
| Cu | picoeukaryotes | A | linear | 0.033 |
| Cu | picoeukaryotes | B | linear | 0.22 |
| Cu | picoeukaryotes | C | linear | 0.078 |
| Cu | picoeukaryotes | D | linear | 0.24 |
| Cu | *Prochlorococcus* | A | quadratic | 0.95 |
| Cu | *Prochlorococcus* | B | quadratic | 0.72 |
| Cu | *Prochlorococcus* | C | logarithmic | 0.7 |
| Cu | *Prochlorococcus* | D | logarithmic | 0.54 |
| Light | *Synechococcus* | A | quadratic | 0.38 |
| Light | *Synechococcus* | B | quadratic | 0.35 |
| Light | *Synechococcus* | C | logarithmic | 0.89 |
| Light | *Synechococcus* | D | logarithmic | 0.93 |
| Light | picoeukaryotes | A | quadratic | 0.66 |
| Light | picoeukaryotes | B | logarithmic | 0.77 |
| Light | picoeukaryotes | C | quadratic | 0.84 |
| Light | picoeukaryotes | D | quadratic | 0.93 |
| Light | *Prochlorococcus* | A | logarithmic | 0.94 |
| Light | *Prochlorococcus* | B | logarithmic | 0.78 |
| Light | *Prochlorococcus* | C | quadratic | 0.49 |
| Light | *Prochlorococcus* | D | quadratic | 0.83 |

Supplemental Figure S1: Depth profiles from September 1, 2020 and October 20, 2020 showing (A) temperature, (B) salinity, (C) fluorescence-derived Chl *a*, and (D) photosynthetically active radiation (PAR).
